# A Regional Reduction in I_to_ and I_KACh_ in the Murine Posterior Left Atrial Myocardium Is Associated with Action Potential Prolongation and Increased Ectopic Activity

**DOI:** 10.1371/journal.pone.0154077

**Published:** 2016-05-05

**Authors:** Andrew P. Holmes, Ting Y. Yu, Samantha Tull, Fahima Syeda, Stefan M. Kuhlmann, Sian-Marie O’Brien, Pushpa Patel, Keith L. Brain, Davor Pavlovic, Nigel A. Brown, Larissa Fabritz, Paulus Kirchhof

**Affiliations:** 1 Institute of Cardiovascular Science, University of Birmingham, Birmingham, United Kingdom; 2 Physical Sciences of Imaging in the Biomedical Sciences, School of Chemistry, College of Engineering Physical Sciences, University of Birmingham, Birmingham, United Kingdom; 3 St George’s, University of London, London, United Kingdom; 4 Department of Cardiovascular Medicine, Hospital of the University of Münster, Münster, Germany; 5 University Hospitals Birmingham NHS Foundation Trust, Birmingham, United Kingdom; 6 Sandwell and West Birmingham Hospitals NHS Trust, Birmingham, United Kingdom; Georgia State University, UNITED STATES

## Abstract

**Background:**

The left atrial posterior wall (LAPW) is potentially an important area for the development and maintenance of atrial fibrillation. We assessed whether there are regional electrical differences throughout the murine left atrial myocardium that could underlie regional differences in arrhythmia susceptibility.

**Methods:**

We used high-resolution optical mapping and sharp microelectrode recordings to quantify regional differences in electrical activation and repolarisation within the intact, superfused murine left atrium and quantified regional ion channel mRNA expression by Taqman Low Density Array. We also performed selected cellular electrophysiology experiments to validate regional differences in ion channel function.

**Results:**

Spontaneous ectopic activity was observed during sustained 1Hz pacing in 10/19 intact LA and this was abolished following resection of LAPW (0/19 resected LA, P<0.001). The source of the ectopic activity was the LAPW myocardium, distinct from the pulmonary vein sleeve and LAA, determined by optical mapping. Overall, LAPW action potentials (APs) were *ca*. 40% longer than the LAA and this region displayed more APD heterogeneity. mRNA expression of *Kcna4*, *Kcnj3* and *Kcnj5* was lower in the LAPW myocardium than in the LAA. Cardiomyocytes isolated from the LAPW had decreased I_to_ and a reduced I_KACh_ current density at both positive and negative test potentials.

**Conclusions:**

The murine LAPW myocardium has a different electrical phenotype and ion channel mRNA expression profile compared with other regions of the LA, and this is associated with increased ectopic activity. If similar regional electrical differences are present in the human LA, then the LAPW may be a potential future target for treatment of atrial fibrillation.

## Introduction

The human left atrial posterior wall (LAPW) has been proposed as a key anchor point for atrial re-entrant activity in atrial fibrillation (AF) [1–3]. The LAPW myocardium has a different embryonic origin to the left atrial appendage (LAA) and pulmonary veins (PVs) [4,5]. Furthermore, in animals, the LAPW exhibits a heterogeneous response to autonomic vagal stimulation [6] and has an increased susceptibility to stretch induced conduction slowing [7], potentially indicative of a unique region-specific electrical identity. Precisely how the electrical phenotype of the LAPW differs from other areas of the LA myocardium is however, currently unresolved. Furthermore, it is unclear whether this area of myocardium (different from the PVs) can generate ectopic action potentials. Here, we therefore compared the electrical properties and ion channel mRNA expression profile of the LAPW with the LAA, to more clearly characterise the regional electrical differences that exist throughout the murine LA myocardium.

We analysed transmembrane action potentials (TAPs) and used optical mapping [8] of the LAA and LAPW to systematically characterise the regional AP properties and to reveal the origins of any spontaneously developing ectopic electrical activity within the LA myocardium. Regional differences in ion channel mRNA expression and I_to_, I_K1_ and I_KACh_ current densities were also assessed.

These experiments show that a significant amount of spontaneous LA ectopy originates from the LAPW myocardium. In addition, the LAPW exhibits prolonged action potential durations (APDs), displays more intra-regional APD heterogeneity and has a distinct ion channel mRNA expression profile. Isolated cells from the LAPW have a significantly reduced I_to_ and I_KACh_ current densities, likely to contribute to the prolonged APD. Thus, the LAPW has a different electrical phenotype compared to other parts of the LA and is more vulnerable to developing spontaneous APs that could promote arrhythmogenesis. If similar findings are validated in humans, antiarrhythmic agents or ablation strategies targeted against LAPW driven ectopy could be a future treatment for AF.

## Methods

### Ethical Statement

All procedures were conducted in accordance with all rules and regulations for experiments with animals and approved by the UK Home Office (PPL number 30/2967) and by the institutional review board of University of Birmingham. Experiments were conducted on male and female adult mice (12–18 week), bred on the MF1 background. Mice were housed in individually ventilated cages, with sex-matched littermates (2–7 mice/ cage), under standard conditions: 12 h light/dark circle, 22°C and 55% humidity. Food and water were available *ad libitum*. The general health status of all mice (bearing, grooming, behavior, body weight) used in the study was monitored daily and immediately prior to surgery. Mouse hearts were extracted by thoracotomy under deep terminal isoflurane anaesthesia (4% isoflurane in O_2_, 1.5L/min), death by exsanguination.

### Left atrial transmembrane action potentials

Following isolation the whole mouse heart was immediately transferred into a dissecting chamber and continuously superfused with a bicarbonate buffered Krebs-Henseleit (KH) solution containing in mM: NaCl 118; NaHCO_3_ 24.88; KH_2_PO_4_ 1.18; Glucose 5.55; Na-Pyruvate 5; MgSO_4_ 0.83; CaCl_2_ 1.8; KCl 3.52, equilibrated with 95%O_2_/5% CO_2_, pH 7.4. Micro-dissection of the LA was performed using a dissection microscope (Stemi SV 11, Zeiss, Germany). The posterior surfaces of the atria were identified, the PV was removed and then the entire intact LA (including the LAA and LAPW) was dissected free by cutting at the junction between the LAPW and the PV orifice.

The LA was transferred into a recording chamber, and pinned onto a Sylgard-coated surface, carefully ensuring not to stretch either the LAA or LAPW. The LA was continuously superfused (KH buffer solution, pH 7.4, 36–37°C, equilibrated with 95% O_2_, 5% CO_2_) and paced at 1–10 Hz via bipolar platinum electrodes. TAPs were recorded from freely contracting LA using custom made glass floating microelectrodes containing 3M KCl, (resistance 15–30 MΩ). Voltage signals were amplified and digitised at 20 kHz and were unfiltered (Axoclamp 2B; Molecular Devices, California, USA; *Spike2* software Cambridge Electronic Design, Cambridge, UK). Measured parameters included the resting membrane potential (RMP), action potential amplitude (APA), peak depolarisation rate (Vmax) and action potential duration (APD) at 30–90% repolarisation. APs were only analysed following sufficient rate adaptation.

To assess for ectopy, TAPs were recorded from the left atrial appendage and LA was stimulated at 1Hz for 3–5 minutes. Ectopic preparations were defined as having more than 2% ectopic APs during 1Hz pacing. All experiments were performed after 15 minutes equilibration and were completed within 2 hours of isolation.

### Optical mapping of activation and action potential duration

Following isolation, murine hearts were mounted on a vertical Langendorff apparatus (Hugo Sachs, March-Hugstetten, Germany). The aorta was retrogradely perfused at 36–37°C, pH 7.4 and loaded with Di-4-ANEPPS (5μM; Biotium, California, USA) for 10–15 minutes. The entire LA was isolated and the PV, inter-atrial septum and coronary sinus were removed from the preparation. The posterior LA surface was exposed in a recording chamber. The uncoupler blebbistatin (10μM; Cayman Chemical, Michigan, USA) was added to the superfusate to minimise contraction artefacts. Following 15 minutes equilibration, the LA was paced (2ms duration pulses, twice diastolic voltage threshold) at 1-10Hz via bipolar platinum electrodes using an isolated constant voltage stimulator (Digitimer, Welwyn Garden City, UK). APs used for analysis were obtained following sufficient rate adaptation [8].

Di-4-ANEPPS was excited at 530nm by four LEDs (Cairn Research, Kent, UK) and emitted fluorescence was captured using a second generation, high spatial resolution (2048 by 2048 pixels, single pixel area: 6.5**μ**m by 6.5**μ**m) ORCA flash 4.0 camera (Hamamatsu Photonics, Japan). Images were recorded and organised using WinFluor V3.4.9 (Dr John Dempster, University of Strathclyde, UK). OAPs (recorded from an ROI of 4x4 pixels) were analysed using custom made algorithms produced in MATLAB [8]. Activation maps were generated as described previously [8].

### Taqman Low Density Array (TLDA) mRNA expression analysis

LA were harvested and dissected into the specific LA regions (LAPW and LAA) before being snap frozen. RNA was isolated from the regional tissue using RNeasy micro kit (Qiagen, Hilden, Germany) with the Precellys Lysis kit containing ceramic beads CK28 (Bertin Technologies, Montigny-le-Bretonneux, France). cDNA was produced using the SuperScript® VILO™ cDNA Synthesis kit (Life Technologies, Paisley, UK). 350ng of RNA was used per TLDA reservoir. Ion channel gene expression levels were quantified using custom-designed and preloaded 96-well TLDA (Life Technologies, Paisley, UK). PCRs were performed using an ABI Real Time PCR 7900HT (Life Technologies, Paisley, UK). Data was acquired using AB SDS 2.4 and RQ manager software (Life Technologies, Paisley, UK). The CT cut off value was 32.

### Left atrial cardiomyocyte cell isolation

Hearts were removed under deep terminal inhalation anaesthesia (4% isoflurane in O_2_, 1.5L/min) and perfused at 4ml.min^-1^ at 37°C on a vertical Langendorff apparatus with the following solutions, equilibrated with 100% O_2_: (i) HEPES-buffered modified Tyrode’s solution containing in mM: NaCl 145, KCl 5.4, CaCl_2_ 1.8, MgSO_4_ 0.83, Na_2_HPO_4_ 0.33, HEPES 5, and glucose 11 (pH 7.4, NaOH) x 5min; (ii) Ca^2+^-free Tyrode’s solution × 5min; (iii) Tyrode’s enzyme solution containing 20**μ**g/mL Liberase™ (Roche, Indianapolis, IN), 0.1% bovine serum albumin (BSA, Sigma), 20mM taurine and 30**μ**M CaCl_2_ × 20-23min. The heart was removed from the Langendorff and perfused with 5ml of modified Kraft-Bruhe (KB) solution containing in mM: DL-potassium aspartate 10, L-potassium glutamate 100, KCl 25, KH_2_PO_4_ 10, MgSO_4_ 2, taurine 20, creatine 5, EGTA 0.5, HEPES 5, 0.1% BSA, and glucose 20 (pH 7.2, KOH). The entire LA was dissected free, separated into LAA and LAPW regions, and cardiomyocytes were dissociated by gentle trituration with fire-polished glass pipettes (2 to 1mm diameter in sequence). Cells were filtered through 100**μ**m nylon gauze and the suspension was centrifuged for 5min at 500–1000 rpm. The remaining pellet was re-suspended in 2ml KB buffer and Ca^2+^ was gradually reintroduced to the cell suspension incrementally over a period of 2 hours to reach a final concentration of 1.8mM. All experiments were performed within 12 hours of isolation.

### Whole cell patch clamp electrophysiology

Dissociated mouse LA cardiomyocytes were transferred to an initially static bath recording chamber and allowed to adhere to laminin-coated coverslips (10mm diameter). Cells were then continually superfused at 3 ml/min, with an external solution containing in mM; NaCl 140, KCl 5.4, CaCl_2_ 1, MgCl_2_ 1, HEPES 10 and glucose 5.5 (pH 7.4 with NaOH). To block L-type Ca^2+^ currents, 300**μ**M CdCl_2_ was added to the superfusate. Experiments were performed at 22±0.5°C. Whole cell patch clamp recordings were obtained in voltage clamp mode using borosilicate glass pipettes (tip resistances 1.5–3 MΩ). For recordings of all K^+^ currents, the pipette solution contained in mM: KCl 135, NaCl 4, EGTA 10, HEPES 10, MgATP 3, Na_3_GTP, 0.5 and glucose 5 (pH 7.2, KOH). Voltage dependent, Ca^2+^ independent K^+^ currents were evoked by 10mV step depolarisations (500ms) from a holding potential of -70mV (close to physiological RMP), at 1Hz. I_to_ was calculated as the difference between peak outward and steady state K^+^ current as described previously [9,10], as I_Kur_ hardly inactivates at 22°C [11]. I_K1_ current was isolated by addition of 50**μ**M BaCl_2_ and applying 10mV step depolarisations (500ms) from -120mV to +50mV from a holding potential of -60mV. Addition of 10**μ**M Carbachol (CCh) to the superfusate in the presence of 50**μ**M BaCl_2_ (that blocks I_K1_ without affecting I_KACh_) was used to maximally activate muscarinic receptors [12]. The CCh dependent current was calculated and used to estimate I_KACh_ as previously described [13]. All recordings and analysis protocols were performed using an Axopatch 1D amplifier (Molecular Devices, USA) and a CED micro1401 driven by Signal v6 software (CED, UK). During experimentation the liquid junction potential (LJP) varied between +3 and +14mV. Best fit I_K1_ and I_KACh_ I/V curves with corrected LJP were calculated post experimentation and were plotted alongside uncorrected values for comparison. The capacitance of each cardiomyocyte was measured by integrating the capacitance current evoked by 10mV depolarising steps from a holding potential of -70mV. The mean cell capacitance was not significantly different between LAA (73±4pF, N = 41) and LAPW (77±3pF, N = 29) cells. Data was only analysed from cells where the input resistance remained above 500MΩ throughout. Series resistance was not compensated, however, voltage errors were assumed to be low given that peak currents were usually less than 2nA.

### Data analysis

Values in text are expressed as mean ± standard error of mean unless otherwise stated. For boxplots, boxes and box limits indicate the median and inter quartile range. All individual measurements are shown in the boxplots as points. Statistical analysis was performed using 1) a Fisher’s exact test 2) a paired 2-tailed student’s t-test, 3) one way repeated measures Analysis of Variance (ANOVA) or 4) two way repeated measures ANOVA with Bonferroni post hoc analysis where appropriate (Prism6, GraphPad, Cal, USA). Significance was taken as two tailed, P<0.05.

## Results

### The LAPW displays intrinsic ectopic activity at low frequency stimulation

**Fig 1:**
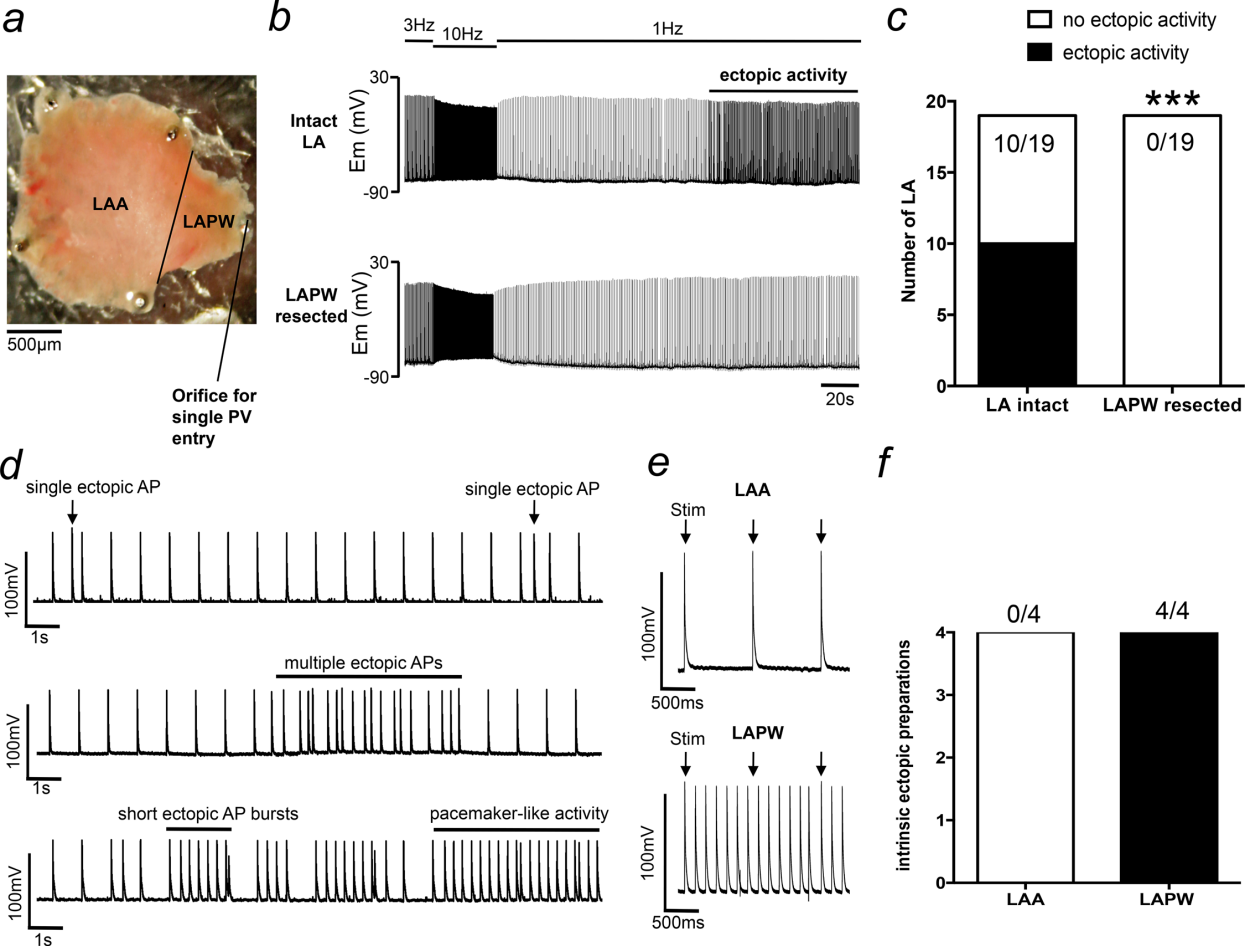
Spontaneous left atrial (LA) ectopy is dependent on intrinsic triggered activity generated in the left atrial posterior wall (LAPW). (a) Light field image of the murine LA showing the anatomical position of the left atrial appendage (LAA) and the LAPW. (b) Example intracellular recordings taken from the same LA with the LAPW attached (intact LA) and after the LAPW has been removed (resected). Ectopic action potentials are abolished following LAPW resection. (c) Number of LA that developed spontaneous ectopic activity before and after LAPW resection. ***denotes P<0.001, Fisher’s exact test, N = 19 LA. (d) Example traces demonstrating different types of ectopic activity. (e) Direct recordings made from the LAA (upper) and LAPW (lower) following LAPW resection. Only the LAPW displays intrinsic ectopic activity. (f) Number of isolated LAA and LAPW preparations that display intrinsic ectopic activity.

Initial experiments aimed to investigate the vulnerability of different LA regions in generating ectopic activity. To do this, intact LAs were paced at 10Hz followed by a more prolonged (up to 5minutes) 1Hz frequency (Fig 1A & 1B). Using this protocol, ectopic activity developed in 10/19 LA preparations (Fig 1B & 1C). The type of ectopic activity varied between preparations and consisted of single isolated ectopic APs, multiple clustered irregular APs and short regular AP bursts that developed into more prolonged pacemaker-like activity (Fig 1D). Interestingly, in these same preparations, all the ectopic activity was abolished after resection of the LAPW (Fig 1B & 1C). In a further 4 ectopic LA preparations, TAP recordings made directly from the resected LAPW tissue showed that it continued to sustain intrinsic ectopic/pacemaker like activity (N = 4/4), whilst the LAA did not (N = 0/4, Fig 1E & 1F). This suggested that the LAPW was the source of the observed ectopy.

**Fig 2:**
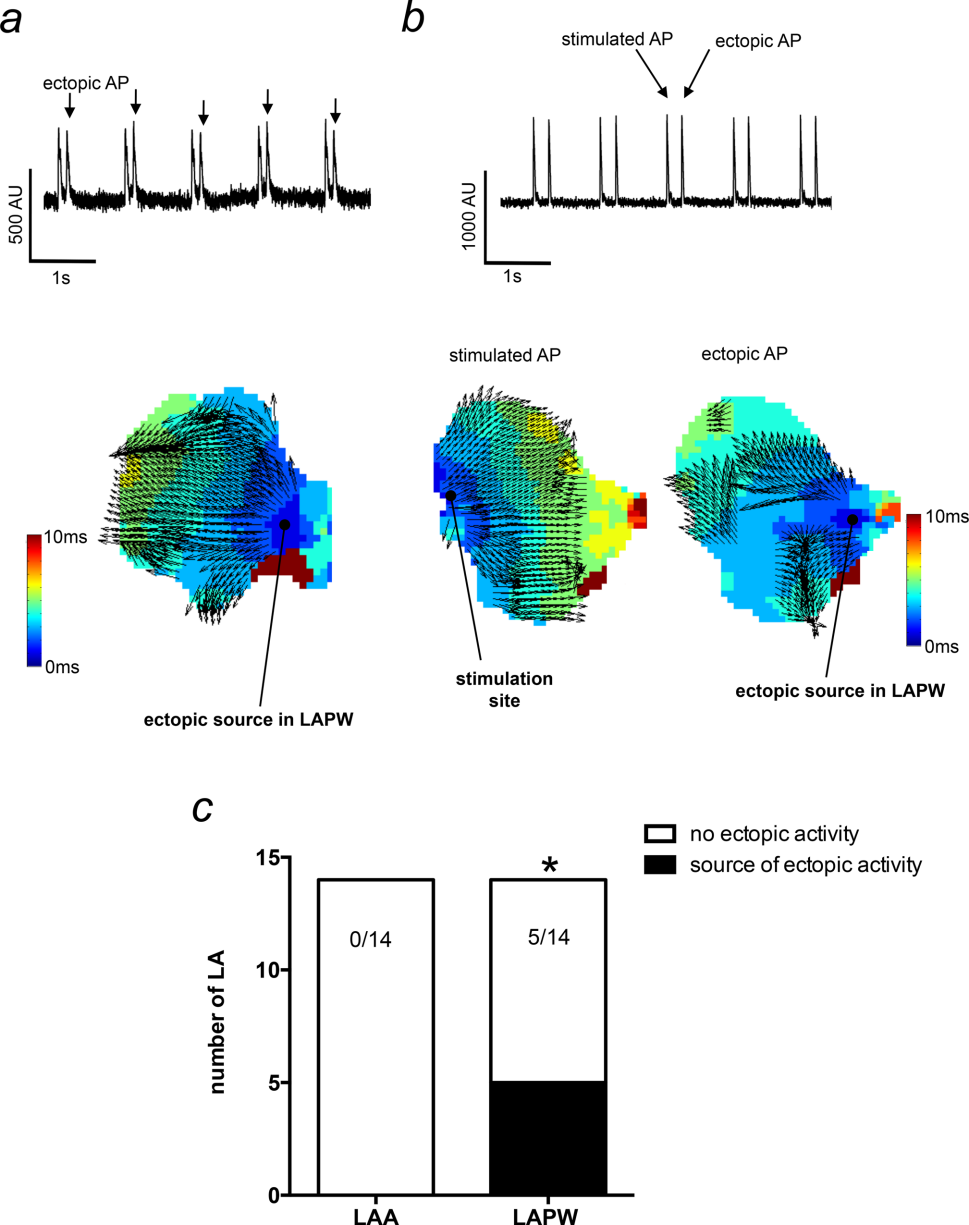
Activation mapping of ectopic action potentials (APs) originating in the left atrial posterior wall (LAPW). (a) Raw voltage sensitive fluorescence trace demonstrating ectopic APs in the LA (upper). The corresponding activation map for the ectopic APs is shown (lower) and illustrates the source of the ectopic AP originating in the LAPW. (b) A raw voltage sensitive fluorescence trace (upper) taken from another preparation that was stimulated in the left atrial appendage (LAA). The stimulated AP activation map originates from the LAA stimulation site (lower left), whilst the ectopic AP originates in the LAPW (lower right). Thus, ectopy is not dependent on close proximity to the stimulus electrode. (c) Number of LA that developed spontaneous ectopic activity with the source in the LAA or LAPW. *denotes P<0.05, Fisher’s exact test, N = 14 LA.

To more clearly define the precise origin of the ectopic activity, we generated isochronal activation maps of the entire intact LA. In these experiments, ectopic activity was found in 5/14 LA. Importantly, in all 5 instances, the source of the ectopic activity was the LAPW myocardium (Fig 2A–2C). To control for the LAPW ectopic activity being a consequence of a close proximity to the stimulus electrode, these experiments were performed with the stimulus electrode positioned at several different sites on the LA surface. Fig 2B demonstrates a stimulated AP originating from the stimulation site in the LAA, whilst the ectopic AP originates in the LAPW, and in this example, it generates a more disorganised propagation wave. Thus, LAPW ectopic activity appears to be independent of the position relative to the stimulus electrode.

### Action potential prolongation and heterogeneity in the LAPW

**Fig 3:**
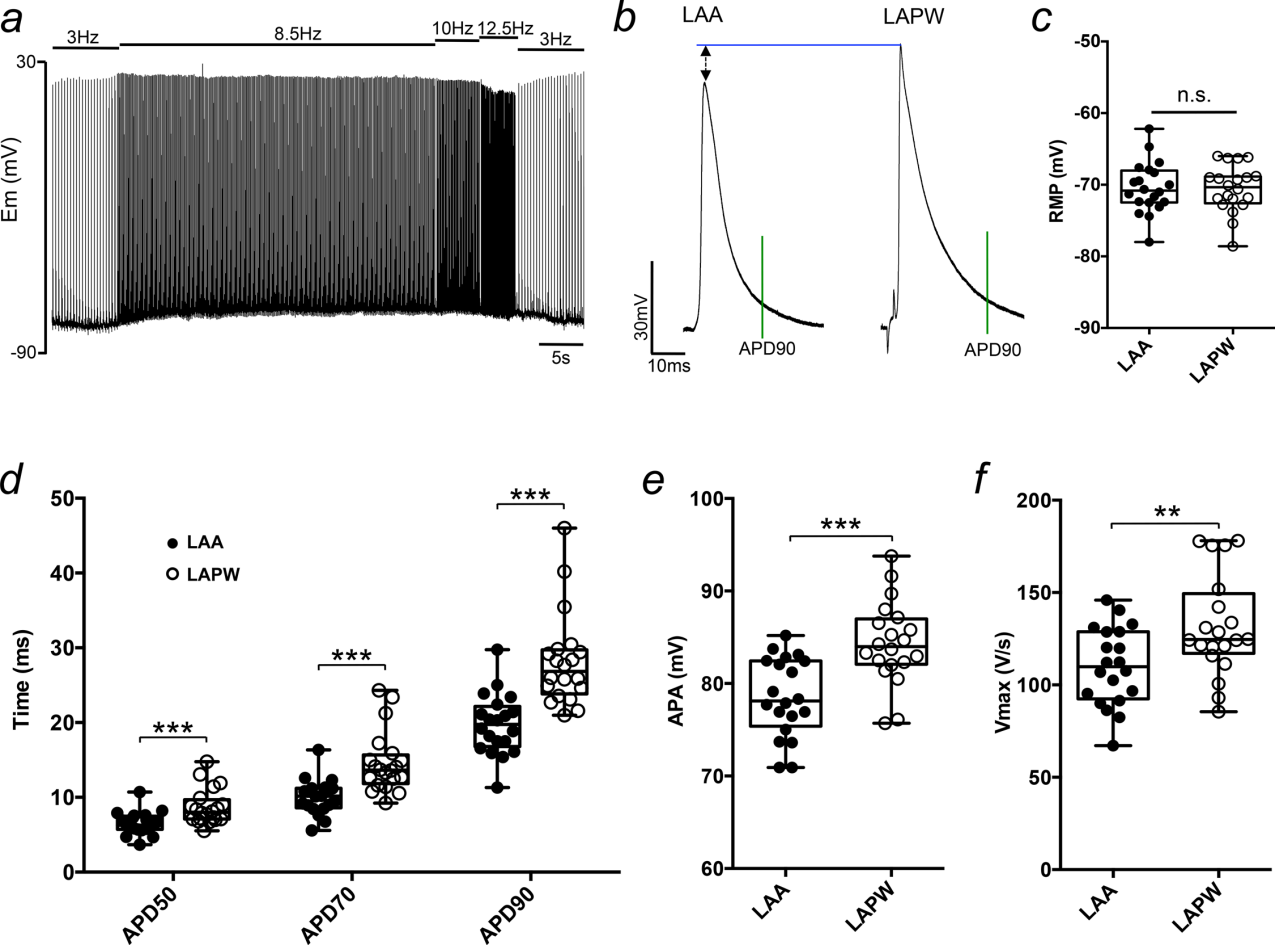
Action potential differences between cardiomyocytes in the left atrial posterior wall (LAPW) and left atrial appendage (LAA). (a) Example intracellular recording trace demonstrating the stimulation protocol used to achieve sufficient action potential rate adaptation. (b) Example transmembrane action potentials (TAPs) taken from the LAA and LAPW of the same left atrium. TAPs are aligned at the resting membrane potential (RMP). The green vertical line indicates action potential duration at 90% repolarisation (APD90). (c-f) Box and whisker plots and individual values comparing the RMP, APD50-90, action potential amplitude (APA) and dV/dt (Vmax), of the LAA and LAPW, at 10Hz pacing frequency. **, *** denotes P<0.01 and P<0.001, LAA v LAPW, one way repeated measures Analysis of Variance (ANOVA) with Bonferroni post hoc analysis, or paired t-test; N = 20 LA.

The ability of the LAPW but not the LAA to generate spontaneous APs implies that there may be fundamental electrical differences between the two regions. To examine potential regional electrical differences in AP morphology, we compared TAPs from the LAA and LAPW using an incremental ramp stimulation protocol consisting of 300 APs at 8.5Hz, 50APs at 10Hz and 50APs at 12.5Hz (Fig 3A). This method allowed for sufficient AP rate adaptation and stabilisation of the AP waveform. At 10Hz, the APD was prolonged (*ca* 40%) in cells from the LAPW compared with cells from the LAA at 50, 70 and 90% repolarisation (Fig 3B & 3D). APD was also longer at 30% repolarisation measuring 4.4±0.2ms for LAA and 5.5±0.3 for LAPW cells (P<0.01, N = 20 LA). Furthermore, the APA and Vmax were significantly greater in LAPW cells by *ca* 10% and 20–25% respectively (Fig 3E & 3F). At 10Hz, the RMP was not different between the LAA and LAPW (Fig 3C). Interestingly, in cells from both regions the RMP did significantly depolarise during high frequency pacing (as exemplified in Fig 3A). This depolarisation was fully reversible (I.E. the RMP returned to the same value after the period of higher frequency pacing as observed before). Importantly, the magnitude of this depolarisation was the same for each region, when calculated as the RMP difference between 3 and 10Hz (LAPW +5±0.5 & LAA +7±1mV, P>0.05, paired 2-tailed student’s t-test). Thus, the enhanced ectopic activity in the LAPW observed in the earlier experiments was probably not the consequence of a build up in extracellular K^+^ brought about during the periods of higher frequency pacing. Overall however, these data do identify electrical differences between LAA and LAPW cells.

**Fig 4:**
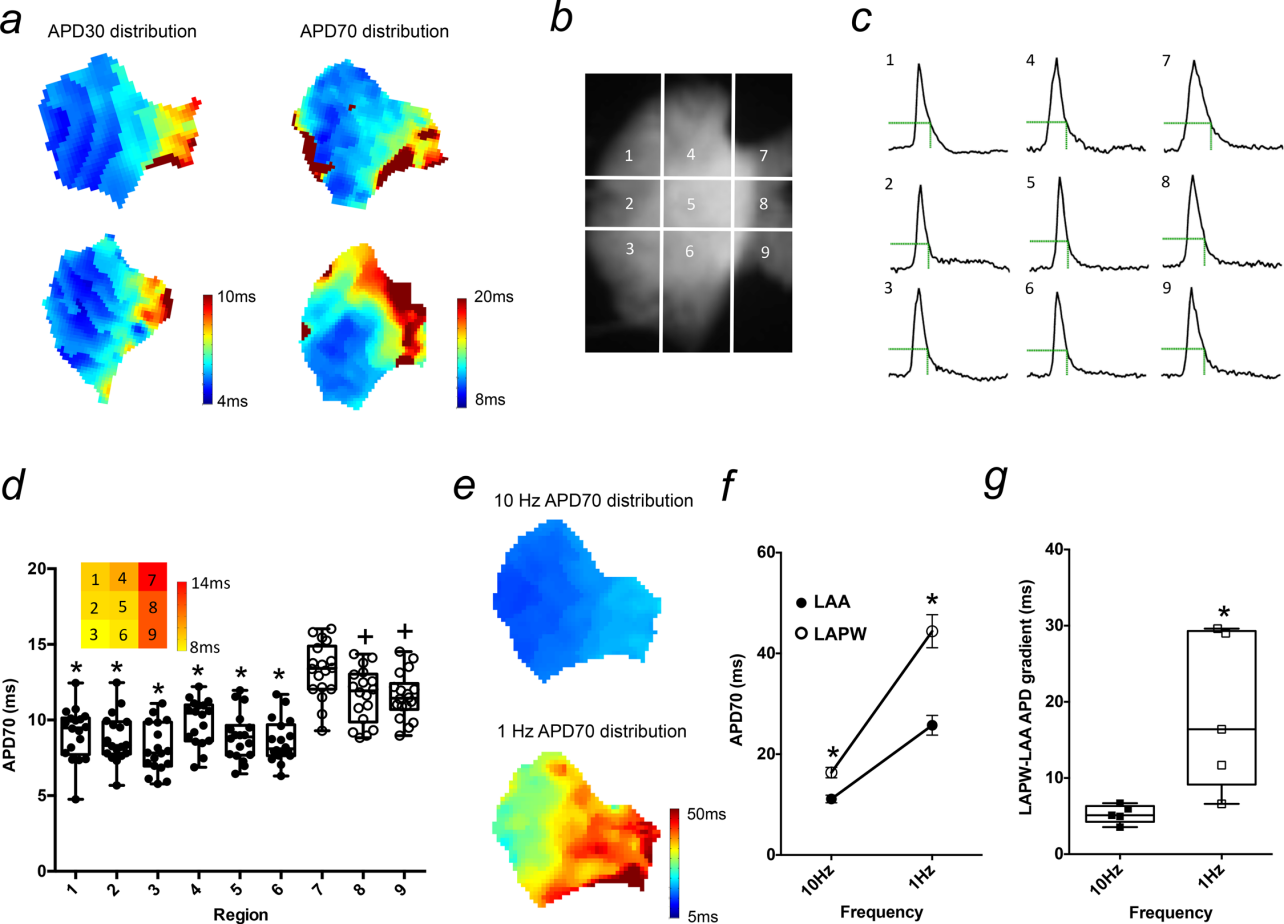
Action potential (AP) prolongation and heterogeneity in the left atrial posterior wall (LAPW). (a) Examples of left atrial (LA) isochronal action potential duration (APD) distribution maps at 30 and 70% repolarisation. (b) A raw fluorescence image of an LA loaded with Di-4-ANEPPS, along with the 9 region grid used for quantitative regional analysis. (c) Example optical action potentials (OAPs) recorded from the 9 different LA regions during 10Hz pacing. The green dotted line indicates APD70. (d) Box and whisker plot of APD70 values measured in each LA region. * denotes P<0.05 vs regions 7,8,9 inclusive, + P<0.05 vs region 7 only, one way repeated measures Analysis of Variance (ANOVA) with Bonferroni post hoc analysis, N = 18 LA. Inset: Heat map depicting mean APD70 values of the 9 LA regions of the LA. (e) Example isochronal APD70 distribution maps of the same LA at 10 and 1Hz (same scale). (f) Mean APD70 at 10 and 1Hz for the left atrial appendage (LAA) and left atrial posterior wall (LAPW). * denotes P<0.05 LAA v LAPW, one way repeated measures Analysis of Variance (ANOVA) with Bonferroni post hoc analysis, N = 5 LA. (g) LA gradients at 10 and 1Hz. * denotes P<0.05 LAA v LAPW, paired t-test, N = 5 LA.

We next wanted to investigate whether the regional LA APD variation was simply confined to a single difference between LAA and LAPW cells, or if there was any further APD variation existing within these two regions. To do this, we used optical mapping to generate APD distribution maps of the entire murine LA (Fig 4A). Two example maps each for APD30 and APD70 (recorded at 10Hz) are shown in Fig 4A and demonstrate not only longer but also a more heterogeneous APD distribution within the LAPW region. To quantify this, optical action potentials (OAPs) were compared between 9 equally spaced quadratic regions covering the entire superficial LA surface (Fig 4B). OAPs in the LAPW (regions 7–9) were longer than all other LA regions (1–6; Fig 4C & 4D). In addition, APD70 was significantly longer in region 7 compared with regions 8 and 9, demonstrating APD heterogeneity within the LAPW that was not apparent in the 6 regions of the LAA (Fig 4C & 4D). Thus, APs in the LAPW were longer than the LAA but also showed a more heterogeneous arrangement of APD.

Since the observed ectopic activity in previous experiments emerged at 1Hz, it was also important to evaluate the regional difference in APD at this slower pacing frequency. As expected, we observed APD lengthening in both the LAA and LAPW with a shift from 10 to 1Hz (Fig 4F). Importantly, the magnitude of rate dependent APD increase was greatly exaggerated in the LAPW (Fig 4F), such that, rather than it being diminished, the overall APD difference between the LAA and LAPW regions was markedly increased at slower pacing (Fig 4E & 4G). Collectively therefore, these data suggest that the emergence of ectopic activity in the LAPW at 1Hz was linked with a more prominent increase in APD in the cells in this region and an exaggeration of the LAA to LAPW APD difference.

### Differences in ion channel gene expression between the LAA and LAPW

**Fig 5:**
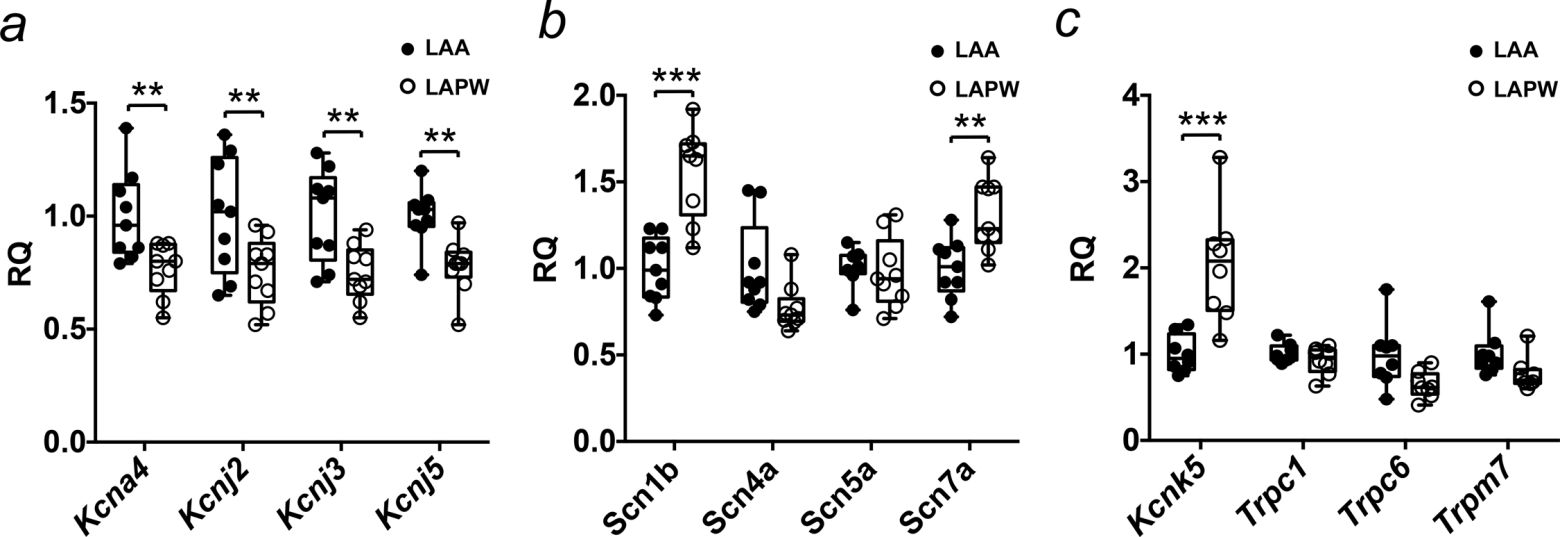
Ion channel expression differences between the left atrial posterior wall (LAPW) and left atrial appendage (LAA). (a-c) Comparisons of K^+^, Na^+^ and background/leak channel gene expression, between the LAPW and LAA, measured using Taqman Low Density Array (TDLA). Control sample was the LAA. ** and *** denote P<0.01 and P<0.001 respectively, LAA v LAPW, paired t-test, N = 9 LA.

To gain insight into the potential molecular causes of the regional variations in AP characteristics we compared the mRNA expression of a panel of 21 ion channel using TLDA (N = 9 LA) in tissue isolated from the LAA and LAPW. Of the K^+^ channel related genes, *Kcna4*, *Kcnj2*, *Kcnj3* and *Kcnj5*, that code for (K_v_1.4, Kir2.1, Kir3.1 and Kir3.4 respectively) were reduced in the LAPW (Fig 5A). *Scn5a*, the most abundant Na^+^ channel alpha subunit gene in the heart, showed no regional differences, while *Scn1b* and S*cn7a* mRNA expression was higher in the LAPW (Fig 5B). In addition, *Kcnk5* mRNA expression; coding for a background/leak channel TASK2 was increased in the LAPW (Fig 5C).

### I_to_ and I_KACh_ are reduced in LAPW cardiomyocytes

**Fig 6:**
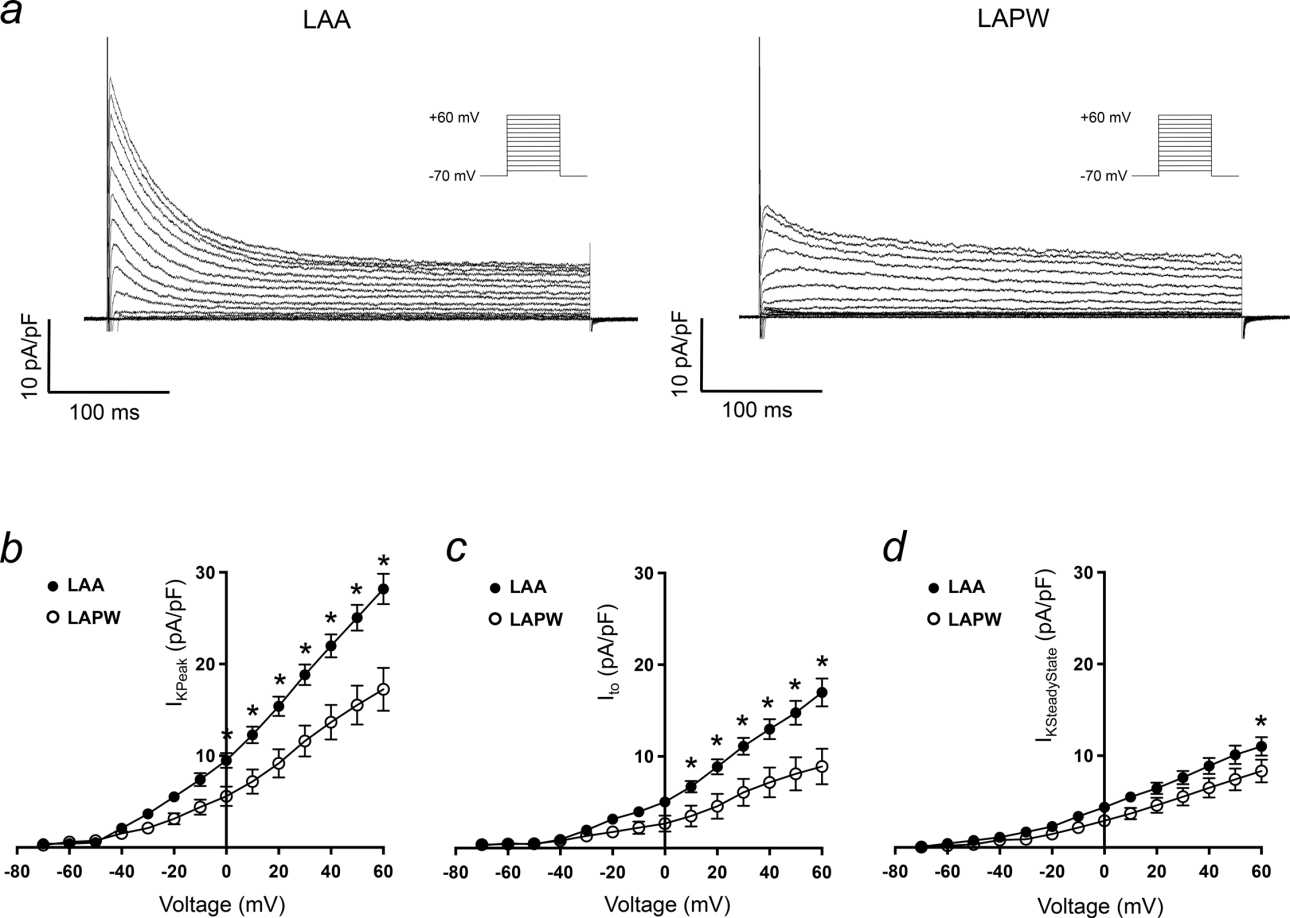
I_to_ reduction in cardiomyocytes isolated from the left atrial poisterior wall (LAPW). (a) Example voltage-sensitive, Ca^2+^ independent, macroscopic K^+^ currents evoked in cardiomyocytes isolated from the left atrial appendage (LAA, left) and LAPW (right). Voltage protocol is shown inset. (b-d) LAA and LAPW I/V relationships for the peak outward K^+^ current, I_to_ and steady state K^+^ current. Data presented as mean ± SEM. * denotes P<0.05 LAA (N = 16 cells) v LAPW (N = 12 cells), two way repeated measures Analysis of Variance (ANOVA) with Bonferroni post hoc analysis.

To examine a potential cause of APD prolongation in the LAPW, measurements of whole cell voltage dependent, Ca^2+^ independent, K^+^ currents were obtained from isolated LAPW and LAA cells (Fig 6A). Peak outward K^+^ current density was significantly reduced in LAPW cells at all voltages positive to 0mV, for example at +20mV; LAA 15±1pA/pF (N = 16 cells) v LAPW 9±1.5pA/pF (N = 12 cells, P<0.05, Fig 6B). I_to_, calculated as the difference between peak and steady state K^+^ at 22±0.5°C [9,10], was reduced in LAPW cells, at all voltages positive to +10mV (Fig 6C). Since I_to_, is an important determinant of atrial repolarisation rate, a marked reduction in the LAPW cells is a good candidate for the observed increase in APD and is consistent with the reduction in *Kcna4* expression. The slowly inactivating/steady state K^+^ current was more similar between the two cell populations, but did show a consistent decrease in LAPW cells, although this was only statistically significant at +60mV (LAA 11±1pA/pF, N = 16 cells v LAPW 8±1pA/pF, N = 12 cells, P<0.05, Fig 6C).

**Fig 7:**
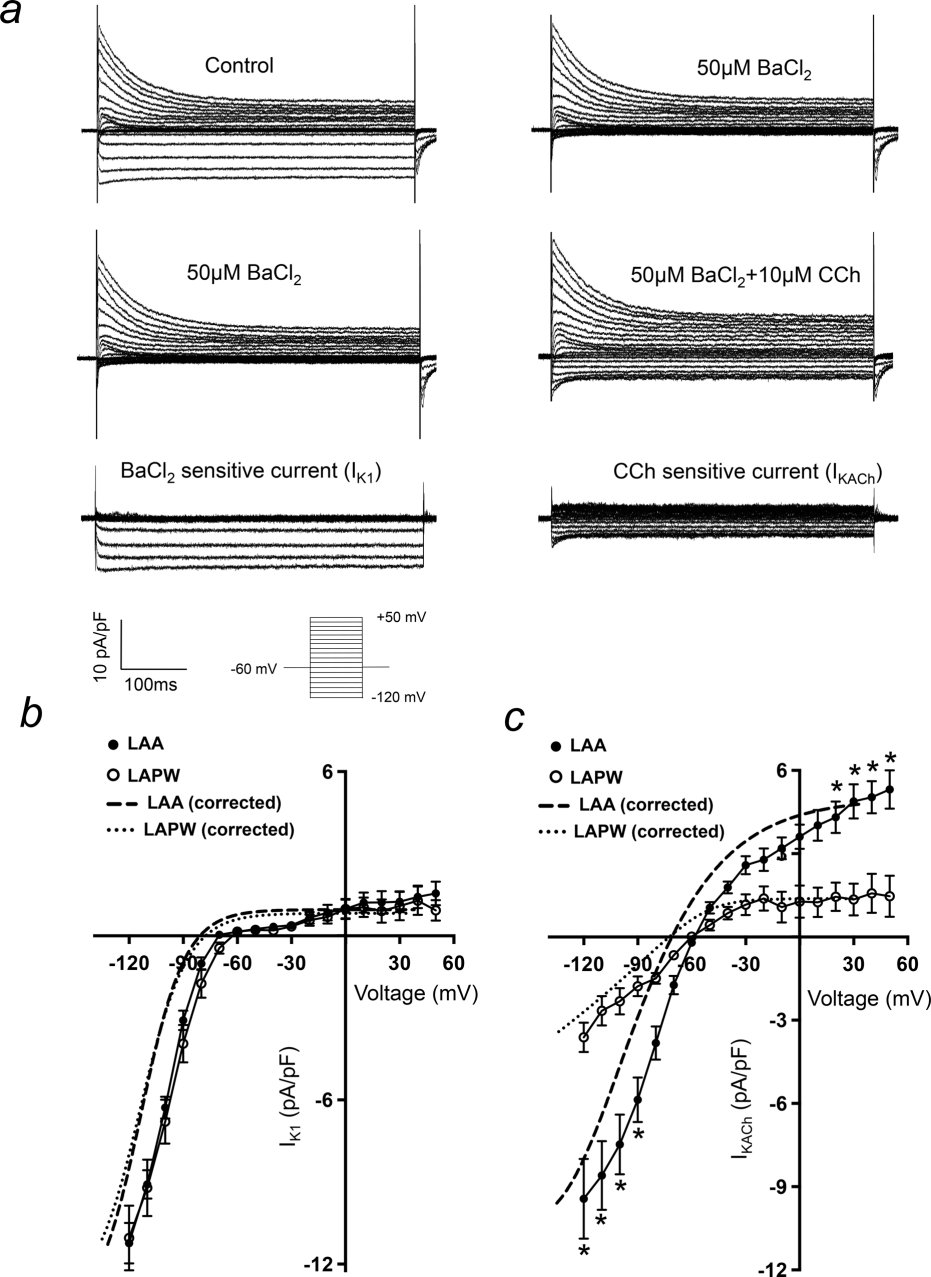
I_KACh_ is depleted in left atrial posterior wall (LAPW) cardiomyocytes. (a) Current traces demonstrating isolation of BaCl_2_ sensitive (I_K1_) and CCh induced (I_KACh_) currents in a single left atrial cardiomyocyte. Voltage protocol is shown inset. (b & c) Comparison of LAA and LAPW I/V relationships for I_K1_ and I_KACh_. The dashed lines indicate mean best fit I_K1_ and I_KACh_ I/V curves with liquid junction potential correction, for both LAA and LAPW. Data presented as mean ± SEM. * denotes P<0.05 LAA (N = 25 cells) v LAPW (N = 17 cells), two way repeated measures Analysis of Variance (ANOVA) with Bonferroni post hoc analysis.

Given that there were observed regional differences in *Kncj2*, *Kcnj3* and *Kcnj5* expression, I_K1_ and I_KACh_ current densities were also compared between the two regions (Fig 7A). I_K1_ current density was equivalent in both LAA and LAPW (Fig 7B), consistent with there being no change in the RMP (see Fig 3C). In contrast, I_KACh_ was significantly depressed in the LAPW, at both negative (-120 to -90mV) and positive (+20 to +60mV) test potentials (Fig 7C).

## Discussion

### Main findings

These data identify some regional electrical differences in the murine left atrium. The LAPW has longer atrial APDs and displays an increased APD heterogeneity compared with the LAA. Furthermore, several key ion channels genes associated with LA repolarisation are decreased in the LAPW including *Kcna4*, *Kcnj3* and *Kcnj5*, and the corresponding ion currents (I_to_ and I_KACh_) are reduced in LAPW cardiomyocytes. The LAPW is also capable of generating spontaneous APs. These findings therefore reveal molecular and functional differences between these two regions of LA myocardium and support the idea that in addition to the pulmonary veins, the LAPW myocardium could be a source of ectopic activity.

### The LAPW has distinct electrophysiological properties

Whilst regional AP variation in the right atrium has been reported in animals [10,14–16] and humans [17], little is known about regional AP variation in the LA, except for a number of studies focusing on the different electrophysiology of PV sleeve [18–21]. More clearly defining the regional electrical differences throughout the LA myocardium may be important in better understanding the different origins and mechanisms of atrial arrhythmia. Here, we demonstrate that cells in the LAPW exhibit larger APA and Vmax than the LAA, consistent with an increased rate of Na^+^ influx. Since the magnitude of depolarising current is an important factor in determining conduction velocity, these findings suggest an accelerated electrical activation spread in the LAPW, consistent with previous studies reporting a fast conduction velocity through this area of myocardium [22]. The observed up-regulation in *Scn1b*, coding for the Na_v_1.5 beta-accessory subunit, is a possible explanation for the increase in Vmax and APA. Whether the change in *Scn1b* expression is causative of the increase in arrhythmia susceptibility observed in the LAPW warrants further consideration. It has been shown that in addition to enhancing peak sodium current density, co-expression of Na_V_1.5 with the beta 1 accessory subunit is also sufficient to cause negative voltage shifts of both activation and steady state inactivation curves [23]. It is also recognised that *Scn1b* modulates the TTX-sensitive sodium current and Ca^2+^ homeostasis in ventricular myocytes [24]. Furthermore, a higher rate of Na^+^ influx may potentially confer the LAPW more susceptible to Na^+^ loading (and thus Ca^2+^ loading). Thus, a detailed comparison of Na^+^ and Ca^2+^ handling between the two regions could provide further insight into better understanding the increased arrhythmia susceptibility of the LAPW region.

We also found prolonged APD in the LAPW. This regional difference correlates with the lower mRNA expression level of *Kcna4* (coding for K_v_1.4) and the significant reduction in I_to_. Although the depressed expression of *Kcna4* is likely to cause a decrease in the slow component of I_to_, we did not attempt to discriminate between I_to,fast_ and I_to,slow_. The finding of APD prolongation in the LAPW at 30% repolarisation, that was subsequently maintained throughout to 90% repolarisation, suggests at least an alteration in a current responsible for early phase repolarisation, for which I_to_ is the best characterised. That said, there may be other currents that are altered in the LAPW and contribute significantly to APD lengthening in addition to those identified here, including I_Kur_, I_CaL_ and I_NaL_.

I_to_ and the fast inactivating component of I_Kur_ have some overlapping time constants, especially at 37°C [11]. Thus, voltage clamp experiments were performed at 22±0.5°C to reduce almost all fast inactivating I_Kur_ thereby allowing for I_to_ to be calculated [11]. There was also a significant reduction in the steady state outward K^+^ current in the LAPW, potentially causing some additional prolongation of the APD.

Despite there being a significant decline in *Kcnj2* expression in the in LAPW, I_K1_ was preserved at all test potentials. This explains the consistency in RMP observed in the 2 regions. The reduction in expression of *Kcnj3* and *Kcnj5*, coding for Kir3.1 and Kir3.4 did associate with a marked depression in I_KACh_. This could account for some further APD prolongation at late phase repolarisation, but whether or not I_KACh_ is constitutively active in the rodent LA remains to be clarified [25,26]. However, regional changes in Kir3.1 and Kir3.4 expression and I_KACh_ do provide a potential explanation for the regional heterogeneous responsiveness of the LA to vagal stimulation previously reported by others [6].

The prolonged APD in the LAPW is an interesting candidate for initiating the observed focal ectopic activity. The APD prolongation was consistent at both early and late phase repolarisation. It is therefore conceivable that APD prolongation could promote some L-type Ca^2+^ channel recovery during late repolarisation thereby leading to Ca^2+^ mediated after depolarisations [27] and extra-systolic activity. Interestingly, the increase in APD in response to the slower rate pacing was greatly exaggerated in LAPW cells, such that the relative APD difference between the LAA and LAPW was increased at 1Hz. This more pronounced delay in repolarisation at 1Hz would further increase the likelihood of any time dependent Ca^2+^ channel recovery and thus it is perhaps not surprising that it was at this frequency that we observed the emergence of ectopic activity within the LAPW myocardium.

Our finding of increased APD heterogeneity in the LAPW region is consistent with higher variability in effective refractory periods in the LAPW region found by others [6]. The increase in APD heterogeneity in this region may also contribute to susceptibility of the LAPW to develop and establish re-entrant circuits following the emergence of triggered activity [27,28]. Overall, our observations suggest that the LAPW, in addition to the PV sleeve, has intrinsic arrhythmogenic properties that may be amenable to specific therapy.

### Embryonic origin of the LAPW–a possible explanation for the distinct electrical properties?

The development of the LAPW myocardium is subtly different to that of the LAA; the LAA forms from an out-pouching of the primary heart field, whereas the LAPW develops from the left posterior second heart field [4,5,22,29,30]. The embryological origin of the LAPW is also different to that of the PVs [4]. However, given the nature by which the PVs are incorporated into the LA [31], it is possible that some LAPW myocardium may also be derived from pulmonary myocardium, the extent of which is unknown. Given our electrophysiology findings and in view of the contrasting embryological origins, it is plausible to suggest that the cells in the LAA and LAPW may also have some different structural properties. Evaluation of the micro-anatomy at the two sites with emphasis on transverse tubule density or the relative density of caveolae should be considered in future investigations.

This study was done in murine LA, and confirmation of our observations in larger animals is needed. The different electrical phenotype of the LAPW cells we observed is likely to be related to its embryological derivation. In terms of the difference in LA morphology between human and mouse, the human LA contains four separate PV connections with a significant smooth walled region between them, whilst there is only a single PV connection in the murine LA [31]. Developmentally however, the two species are relatively similar, in that the early human heart has a solitary PV connection, which is only later remodelled to “draw in” the four PVs into the LAPW [31].

### Implications for initiation of AF

The current findings illustrate that the LAPW myocardium (outside of the PVs) is susceptible to developing spontaneous ectopic activity. Importantly, these findings were observed following removal of the PVs and coronary sinus from the preparation, as both have been previously been shown to generate triggered activity [32–34]. Optical mapping confirmed an origin of the observed ectopy in the LAPW. Our data suggest that the LAPW has distinct electrical properties. If confirmed in larger animals and patients, our findings provide a reasonable basis to develop antiarrhythmic drugs targeting the ion channels responsible for APD prolongation in the LAPW surrounding the PVs.

### Conclusion

We show that the LAPW myocardium has an intrinsic capability for the generation of ectopic APs. In addition, there are important electrophysiological differences between the LAPW, originating from the second heart field, and LAA originating from the primary heart field. Prolonged APD, increased APD heterogeneity, reductions in I_to_ and I_KACh_ and differential mRNA expression of ion channel genes associated with AF are possible contributors to the arrhythmogenicity of the LAPW. Our findings will inform further studies characterising the specific role of the LAPW for the genesis, recurrence, and future treatment of AF.
